# Decursinol angelate relieves inflammatory bowel disease by inhibiting the ROS/TXNIP/NLRP3 pathway and pyroptosis

**DOI:** 10.3389/fphar.2024.1520040

**Published:** 2025-01-07

**Authors:** Yudi Wang, Jiamin Wang, Yonghu Chen, Xuezheng Li, Zhe Jiang

**Affiliations:** ^1^ Department of Pharmacy, Yanbian University Hospital, Yanbian University, Yanji, China; ^2^ Key Laboratory of Natural Medicines of the Changbai Mountain, Ministry of Education, College of Pharmacy, Yanbian University, Yanji, Jilin, China

**Keywords:** angelica gigas nakai, NLRP3 inflammasome, IBD, pyroptosis, decursinol angelate

## Abstract

**Introduction:**

Despite evidence of the efficacy of decursinol angelate (DA), a prescription medication derived farom traditional Chinese medicine, in alleviating inflammatory bowel disease (IBD), the precise mechanisms behind its action remain unclear.

**Methods:**

Lipopolysaccharides (LPS) and dextran sodium sulfate (DSS) induction were used as *in vitro* and *in vivo* models of IBD, respectively, to assess the role of DA in alleviating IBD. Enzyme-linked immunosorbent assay (ELISA) was performed to detect the expression levels of pro-inflammatory cytokines in mouse serum, Western blot was performed to detect the expression of TXNIP/NLRP3 pathway tight junction (TJ) proteins in colon tissues and cells, and immunohistochemistry, immunofluorescence and immunohistochemistry, immunofluorescence and qRT-PCR were used to validate the proteins related to this signaling pathway. Molecular docking technique and co-immunoprecipitation (Co-IP) method assay were applied to evaluate the targeting effect of DA on NLRP3 proteins, and MCC950, a specific inhibitor of NLRP3, was used as a positive control for validation.

**Results:**

Our research indicates that DA’s distinctive molecular mechanism could entail binding to the NLRP3 protein, thereby suppressing the activation of the NLRP3 pathway and diminishing the assembly and activation of the NLRP3 inflammasome, thus functioning as an anti-inflammatory agent.

**Conclusion:**

DA may play a role in improving BD by inhibiting the activation of the ROS/TXNIP/NLRP3 signaling pathway and the release of inflammatory mediators, and by repairing the intestinal barrier function.

## 1 Introduction

IBD is a chronic, relapsing inflammatory condition of the gastrointestinal tract with no known specific cause. This disease is characterized by its persistence, tendency to recur, and a multitude of treatment options, yet it remains incurable. The two primary forms of IBD are ulcerative colitis (UC) and Crohn’s disease (CD) (Kobayashi et al., 2020). Research has shown that the origins and progression of IBD are complex, involving a multitude of factors that contribute to the onset and advancement of the disease (de Souza and Fiocchi, 2016).

IBD is influenced by the nucleotide-binding oligomerization domain-like receptor protein 3 (NLRP3) inflammasome both during its initiation and development (Tourkochristou et al., 2019). Reactive oxygen species (ROS) is an oxidative metabolite produced by mitochondria, and excess ROS can activate the NLRP3 inflammasome. Thioredoxin-interacting protein (TXNIP) is an oxidative stress regulator. Research has demonstrated that ROS causes TXNIP to bind to NLRP3, which in turn promotes the endothelium inflammasome’s activation. According to Abais et al. (2015), elevated ROS expression raises TXNIP expression, which encourages NLRP3 activation and causes inflammation.

Biological, chemical, physical, and immunological barriers make up the intestinal barrier. In addition to keeping pollutants and harmful bacteria out of the body, these barriers work together to promote the best possible absorption and use of nutrients (Lee et al., 2018). The progression of inflammatory bowel illness has been linked to an imbalance of biological and physical barriers, including a loss of tight junctions between epithelial cells, an increase in pathogenic bacteria, and a decrease in microbial diversity (Kumar and Kumar, 2022). These findings imply that avoiding IBD may be accomplished by focusing on the intestinal barriers.

**FIGURE 1:**
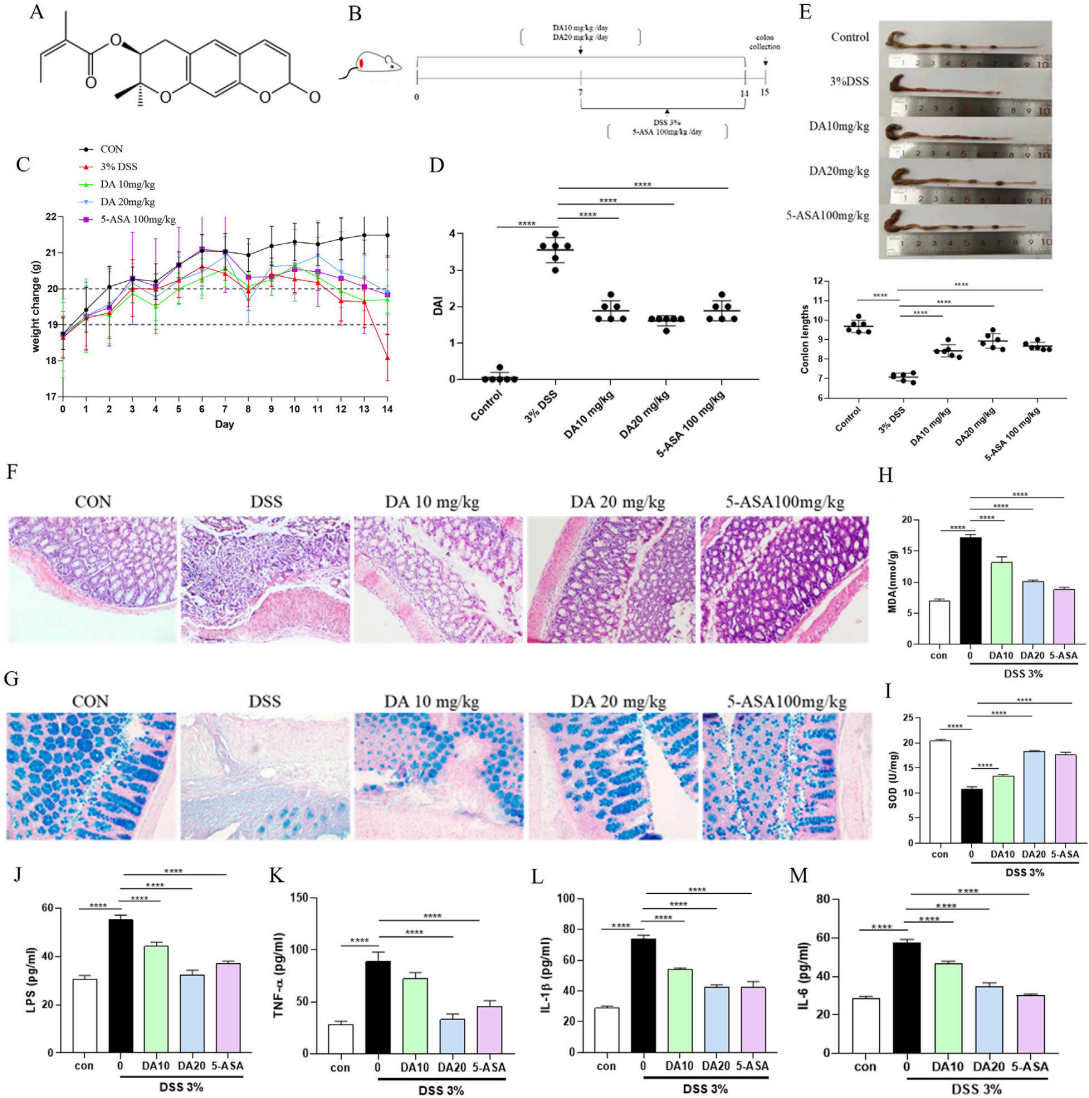
DA treatment alleviates DSS-induced colitis in mice. **(A)** Chemical structure of DA. **(B)** Schematic diagram of the treatment groups and timing of the experiment. **(C)** Body weight changes in each group (n = 8). **(D)** DAI scores in each group. **(E)** Representative photographs of mouse colons and measurements of colon length in each group. **(F)** Results of H&E staining of colon tissues and histological injury scores of mice in each group (200×). **(G)** Representative images of AB staining of colon tissues in each group (200×). **(H, I)** The concentrations of MDA and SOD in the mice’s serum for each group (n = 3). **(J–M)** Results of Inflammatory Factors (IL-1β, IL-6, LPS, and TNF-α) in Serum of Each Group of Mice (n = 3). ****P < 0.0001.

Numerous natural compounds have anti-inflammatory qualities (El-Abhar et al., 2008), may have some antioxidative effects (Kotakadi et al., 2008), and provide protection against exogenous antigenic chemicals. According to Wong et al. (2008), there is a strong correlation between these outcomes and oxidative stress prevention. *A. gigas* Nakai is used in traditional medicine to treat anemia and gynecological disorders, and it also has anti-inflammatory and analgesic properties (He et al., 2023). Decursinol angelate (DA) is a pyranocoumarin-like compound isolated from *A. gigas* Nakai (Figure 1A), which studies have shown to treat intestinal inflammation, pain, anemia, and infections (Ahn et al., 2008). DA has been shown in numerous experiments to suppress mitogen-activated protein kinase (MAPK) activation, the nuclear factor κB (NF-κB) translocation, and the production and exogenous secretion of interleukin (IL)-1β and IL-6 (Islam et al., 2018). The results of DA’s single-layer model of Caco-2 cells indicate that transport is primarily in the intestine (Shehzad et al., 2018). Therefore, in this study, we aimed to explore the possible mechanisms by which DA alleviates IBD both *in vivo* and *in vitro*. We anticipate that our findings will provide a basic framework and scientific reference for the clinical application of DA.

## 2 Materials and methods

### 2.1 Animal model

Forty male 6-week-old (body weight: 20 ± 2 g) BALB/c mice were purchased from the Laboratory Animal Research Center of Yanbian University. They were housed in a temperature-regulated chamber within a specifically pathogen-free environment. The protocol for the animal experiments employed in this study was approved by the Animal Ethics Committee of Yanbian University Hospital (2021199). The mice were randomly divided in the following five treatment groups (n = 8 mice/group): control (CON), DSS (DSS 3%), DSS + 10 mg/kg DA (DA10 mg/kg), DSS + 20 mg/kg DA (DA10 mg/kg), and DSS + 100 mg/kg 5-amino salicylic acid (5-ASA100 mg/kg). In the DA10 mg/kg and DA10 mg/kg groups, mice were administered DA by gavage once a day, starting on day 1, for 14 consecutive days, while the rest of the groups were given phosphate-buffered saline (PBS) solution. In the 5-ASA100 mg/kg group, mice received one daily gavage treatment of 5-ASA starting on day 7, for 7 consecutive days. On the 7th day, the control group started to drink water freely, and the other groups of mice drank 3% DSS solution freely, which was changed daily for 7 consecutive days. The mice were euthanized on the 15th day, and their colon tissues were extracted for subsequent analysis.

### 2.2 Cell culture and treatment

The rat small intestinal crypt epithelial cell line IEC-6 cells were donated by the Chinese Academy of Science (Kunming, China). The cells were cultured in DMEM (Keygen, Nanjing, China) medium supplemented with 10% fetal bovine serum (GeminiBio, CA, United States) at 37°C in a humidified incubator with 5% CO_2_.

In the course of the experiment, the classification scheme utilized was as follows: post-collection, IEC-6 cells were seeded and incubated overnight. Subsequently, they were subjected to a 24-h treatment with 10 μg/mL LPS and varying concentrations of DA (1, 5 µM). Following this, the cells and supernatants were separated for further analysis. Four distinct sets of experimental cells were established and cultivated: the control group (CON), the LPS group (10 μg/mL), the DA 1 µM group (DA 1 µM + LPS 10 μg/mL), and the DA 5 µM group (DA 5 µM + LPS 10 μg/mL).

### 2.3 Assessment of disease activity index (DAI)

The DAI is an important measure of disease severity. Mice were weighed and observed for disease status daily, and physiological indices were recorded. Scores were assigned as follows: (1) In short, weight loss was scored as: 0%–1%, 1%–5%, 5%–10%, 10%–20%, and >20%. (2) N normal stool consistency (0 points), (2) loose stools (2 points), (3) watery diarrhea (4 points), and (3) gross rectal bleeding (0 points), (2) minor bleeding (2 points), and (4) significant bleeding (4 points). After adding up these scores, they were split by three.

### 2.4 Levels of the superoxide dismutase (SOD) and malondialdehyde (MDA)

Following the manufacturer’s instructions, kits from Nanjing Jiancheng, Jiangsu, China were used to assess the serum levels of MDA and SOD. With the use of a Spark microplate reader, the absorbance at 532 and 550 nm was determined.

### 2.5 Enzyme-linked immunosorbent assay (ELISA)

As directed by the manufacturer, serum levels of IL-1β, IL-6, LPS, and TNF-α were measured using ELISA kits (Jiangsu Meibiao, Jiangsu, China). The absorbance at 450 nm was recorded using the Spark microplate reader.

### 2.6 Histological analysis

Mouse colons were fixed in 10% formalin for 72 h, and embedded in paraffin, sectioned into 4 μm slices. The sections were then stained with hematoxylin-eosin (H&E) staining and Alcian blue (AB) staining. H&E staining was used to analyze the general morphologic changes in the colonic tissues, while AB staining was used to assess the level of the mucus layer of the tissues. Representative tissue structures were selected and photographed.

### 2.7 Western blotting

Total proteins were extracted from mouse colon tissues and IEC-6 cells using the lysis buffer PMSF (phenylmethanesulfonyl fluoride) (Solarbio, Beijing, China). Protein concentrations were measured using a bicinchoninic acid (BCA) assay kit (Solarbio, Beijing, China). Proteins in equal quantities were subsequently put onto polyacrylamide-sodium dodecyl sulfate gels for electrophoresis separation. The proteins were subsequently transferred onto PVDF membranes, which have a thickness of 0.22 μm. Membranes were blocked with 5% nonfat dry milk for an hour, and then the appropriate primary antibodies (1:1,000) were incubated at 4°C. The membranes were then treated at room temperature for 2 hours with secondary antibodies (1:20,000). Protein bands were detected using a gel imager (ProteinSimple, United States), and ImageJ 8.0 was employed to analyze the results in grayscale.

### 2.8 Quantitative reverse transcription-PCR (qRT-PCR)

Following the manufacturer’s instructions, total RNA was extracted from colonic tissues and IEC-6 cells using TRIzol reagent (Sigma, Saint Louis, MO, United States). Thermo Scientific, United States NanoDrop spectrophotometer was used to measure the amount and purity of the RNA samples. The isolated RNA was then used to create cDNA using a FastKing cDNA synthesis kit (Tiangen, China). The thermocycling settings were as follows: 15 min at 95°C, 40 cycles of 10 s at 95°C, and 20 s at 60°C. To calculate the relative expression of the genes, the 2^−ΔΔCT^ technique was employed. In this case, the reference gene was GAPDH (Table 1).

**TABLE 1:** Primer sequence information

Gene	Primers (5′-3′)	
NLRP3	TAC​GGC​CGT​CTA​CGT​CTT​CT	Forward Primer
	CGC​AGA​TCA​CAC​TCC​TCA​AA	Reverse Primer
Caspase-1	TCC​AGG​AGG​GAA​TAT​GTG​GGA	Forward Primer
	CAC​CAC​TCC​TTG​TTT​CTC​TCC	Reverse Primer
ASC	GAC​AGT​ACC​AGG​CAG​TTC​GT	Forward Primer
	AGT​CCT​TGC​AGG​TCA​GGT​TC	Reverse Primer
IL-1β	GCC​ACC​TTT​TGA​CAG​TGA​TGA​G	Forward Primer
	CCT​GAA​GCT​CTT​GTT​GAT​GTG​C	Reverse Primer
GAPDH	TGA​CCT​CAA​CTA​CAT​GGT​CTA​CA	Forward Primer
	CTT​CCC​ATT​CTC​GGC​CTT​G	Reverse Primer

### 2.9 Immunohistochemical assessment

The colonic tissue paraffin sections underwent deparaffinization and rehydration to facilitate the detection of NLRP3, gasdermin D (GSDMD), and TXNIP. Subsequently, the samples were immersed in citric acid to execute the antigen retrieval process. The tissues were subsequently coated with 5% BSA and permitted to seal for 1 hour at ambient temperature. Following this, the sections were evenly distributed within a humid chamber and the primary antibody was administered, allowing the tissues to be immunostained overnight. After the sections had been agitated and dried, they were treated with a secondary antibody from the same species as the primary antibody. The next phase involved incubation at room temperature. DAB was then applied to the slices to develop color, and hematoxylin was utilized for counterstaining. Under a microscope, all stained samples were visible.

### 2.10 Cell viability assay

Using the MTT assay (Solarbio, Beijing, China), cell viability was determined. Each well received 0.5 mg/mL MTT solution, which was then left to incubate for 4 h. At 490 nm in wavelength, the sample was measured with a MicroTek Instrument microplate reader. The results were expressed as a viability percentage.

### 2.11 Intracellular ROS measurement

ROS was measured using the 2′,7′-dichlorofluorescein diacetate (DCFH-DA, Beyotime, Beijing, China). After being gathered, IEC-6 cells were plated overnight at a density of 3 × 10^5^ cells per well on 6-well plates. The cells were subjected to 24 h of treatment with 10 μg/mL LPS and varying doses of DA (1, 5 µM). A colorless serum-free DMEM solution was used to dilute the DCFH-DA to a concentration of 10 μg/mL. One milliliter of the DCFH-DA dilution was then applied to each well of the cell and incubated for 15 min at 37°C. Three rounds of DMEM media without serum were used to wash the cells. After that, a fluorescence microscope was used to see and evaluate the fluorescence intensity.

### 2.12 JC-1 assay

IEC-6 cells were collected and then plated on 6-well plates for an overnight period at a density of 3 × 105 cells per well. For 24 h, cells were exposed to LPS and various DA doses (1, 5 µM). Following a single PBS wash, add 1 mL of JC-1 dyeing solution to each well as directed, incubate for 20 min in the cell incubator, and then repeat the washing process with JC-1 dyeing buffer twice. The fluorescence intensity was then seen and assessed using a fluorescence microscope.

### 2.13 Flow cytometry

IEC-6 cells were collected and then plated on 6-well plates for an overnight period at a density of 3 × 105 cells per well. For 24 h, cells were exposed to LPS and various DA doses (1, 5 µM). Following instructions from MBI, Beijing, China, cells were harvested, cleaned, and then resuspended in 500 µL of binding buffer. Annexin V-FITC and propidium iodide (PI) dyes were then added and mixed with each cell sample. After guidelines provided by MBI, Beijing, China, cells were isolated, cleansed, and reconstituted in 500 µL of binding buffer. Each cell sample was then combined with annexin V-FITC and propidium iodide (PI) dyes. The cells were incubated at room temperature for 25 min before being exposed to flow cytometry analysis.

### 2.14 Measurement of lactate dehydrogenase (LDH) content

Cells were collected and spread evenly in 96-well plates, including LDH-positive control wells and LDH-negative control wells containing culture medium only. After 24 h of drug intervention, 100 μL of LDH-releasing reagent was added to each well, mixed well, and the LDH assay working solution was added, followed by a 1-h incubation. The liquid in each well was measured for absorbance at 490 nm after the incubation.

### 2.15 Immunofluorescence analysis

Cells were collected and spread evenly in 6-well plates. After 24 h of drug intervention. Paraformaldehyde for 15 min. The treatment was treated with 5% triton permeation for 15 min. After that, the cell was sealed for 1 hour at room temperature using BSA. The matching primary antibodies are then incubated at 4°C for the entire night. One hour was spent incubating the secondary antibody at room temperature. After assigning a blue color to the DAPI dye channel, the anti-fluorescence quenching reagent was administered, and a fluorescence microscope was used to examine and assess the fluorescence intensity.

### 2.16 Co-IP

Configure the Co-IP cracking liquid according to the instructions, lysed cells, and protein quantification. After adding NLRP3 antibody (1:700) and IgG to the negative control group, the mixture was stirred at 4°C for the whole night. After the magnetic bead treatment, the room temperature shaking bed was 1 h, so that the magnetic bead and antibody fully combined. The magnetic beads were fully cleaned, added with 80 uL water and 20 μL 5 × SDS Buffer, heated at 100°C for 5 min, cooled to room temperature, magnetically separated on a magnetic rack, and the super serum was taken for protein Western blot detection.

### 2.17 Molecular docking

2D molecular structure in compound database PubChem, energy minimization and coordination preparation by Chem 3D, download the crystal structure of key target NLRP3 from Protein Data·Bank (PDB). After the above-treated receptors and ligands were operated by Auto Dock Tools for water removal, hydrogenation, and charge calculation, Auto Dock Vina was used for calculation, and the docking results were visualized and analyzed by PyMOL software. The binding energy is directly correlated with the strength of the receptor-ligand interaction. The lower the binding energy, the higher the degree of mutual adaptation and matching between the two, and the more stable the conformation. The common consensus is that a chemical has good binding activity with the target when the binding energy is ≤ −5.0 kcal/mol.

### 2.18 Statistical analysis

GraphPad Prism 7.0 was used for one-way analysis of variance comparisons of group data. For each experiment, a minimum of three sets of data were gathered. Data are presented as means ± standard deviation. *P*-value < 0.05 was considered to indicate statistical significance.

## 3 Results

### 3.1 DA treatment alleviates DSS-induced colitis in mice

The experimental design and pathological impacts of DA on IBD model mice are shown in Figure 1. Following the administration of this DSS solution in drinking water, the study employed 5-ASA (100 mg/kg) as a positive control, as depicted in Figure 1B. Weight loss is an important indicator of IBD, and DSS-induced mice showed significant weight loss. Therefore, the weight of each group of mice was monitored throughout the process. Treatment with DSS alone led to severe body weight loss, whereas treatment with either dose of DA, or 5-ASA mitigated the loss of body weight (Figure 1C). Compared with the control mice, the DSS mice had an elevated DAI index and a significantly shorter colon, whereas the DA and 5-ASA mice showed a reduction in these symptoms (Figures 1D, E). Additionally, the results of H&E revealed that the treatment of DA reduced the inflammatory alterations caused by DSS, including submucosa edema, immune cell infiltration, crypt loss, and erosion of the epithelial monolayer (Figure 1F), and AB staining showed that mucus decreased in the tissues after DSS induction and increased significantly after DA as well as 5-ASA treatment, and the effect of DA at high doses was comparable to that of 5-ASA (Figure 1G).

MDA and SOD levels associated with oxidative stress were detected in serum, SOD level decreased and MDA level increased in serum of mice induced by DSS, which was inhibited by remission after DA 10 and 20 mg/kg, and 5-ASA 100 mg/kg treatment (Figures 1H, I). ELISA was applied to detect inflammatory cytokines in the serum, we found that both DA and 5-ASA could reduce IL-1β, IL-6, LPS, and TNF-α generation (Figures 1J–M).

### 3.2 DA inhibits activation of TXNIP/NLRP3 signaling pathway in IBD mice

**FIGURE 2:**
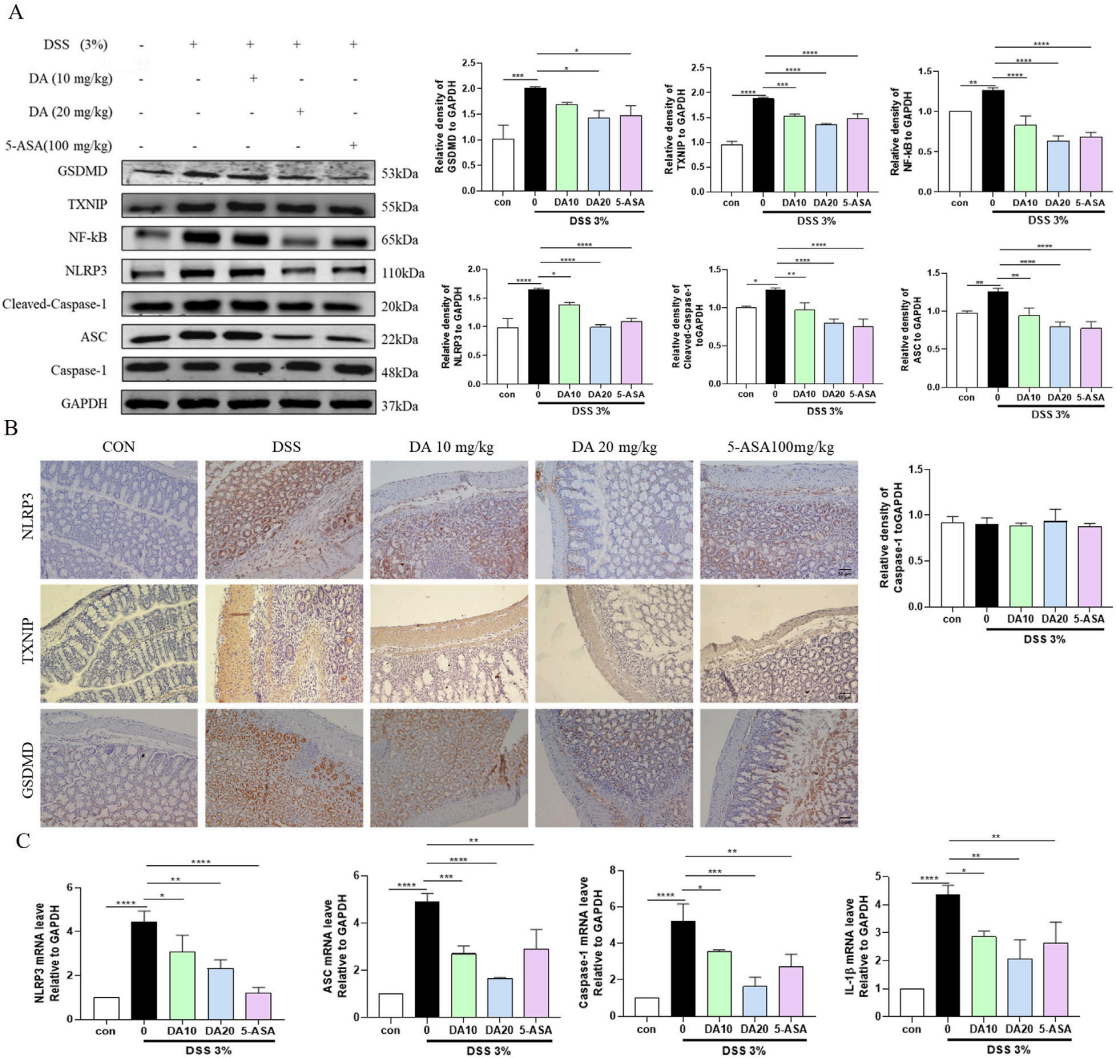
DA restrains the TXNIP/NLRP3 pathway. **(A)** Western blotting and quantitative analysis of TXNIP/NLRP3 pathway-related protein expression in IBD mice. **(B)** Representative immunohistochemical staining images of NLRP3, TXNIP, and GSDMD in colon tissue sections from each group. **(C)** Expression levels of TXNIP/NLRP3 pathway-related mRNA in colon tissues (n = 3). *P < 0.05, **P < 0.01, ***P < 0.001 and ****P < 0.0001.

Intestinal epithelial cells in both IBD patients and the murine experimental model had activated NLRP3 inflammasomes, and the pathophysiology of IBD is also influenced by NLRP3 activation. The expression of the TXNIP, NF-κB, GSDMD, NLRP3, apoptosis-associated speck-like protein containing a CARD (ASC), and Cleaved-cysteinyl aspartate specific proteinase (Caspase)-1 proteins was significantly elevated in colon tissues of DSS-treated mice, but inhibited by DA and 5-ASA (Figure 2A). In addition, the mRNA levels of NLRP3, ASC, Cleaved-Caspase-1, and IL-1β were significantly increased in DSS-induced mouse colon tissues, whereas DA could downregulate the related mRNA levels (Figure 2C). Immunohistochemical results showed that DA and 5-ASA decreased the production of NLRP3、TXNIP and GSDMD in the DSS-treated mice colonic tissue (Figure 2B).

### 3.3 DA therapy reinstates the breakdown of the intestinal barrier caused by DSS

**FIGURE 3:**
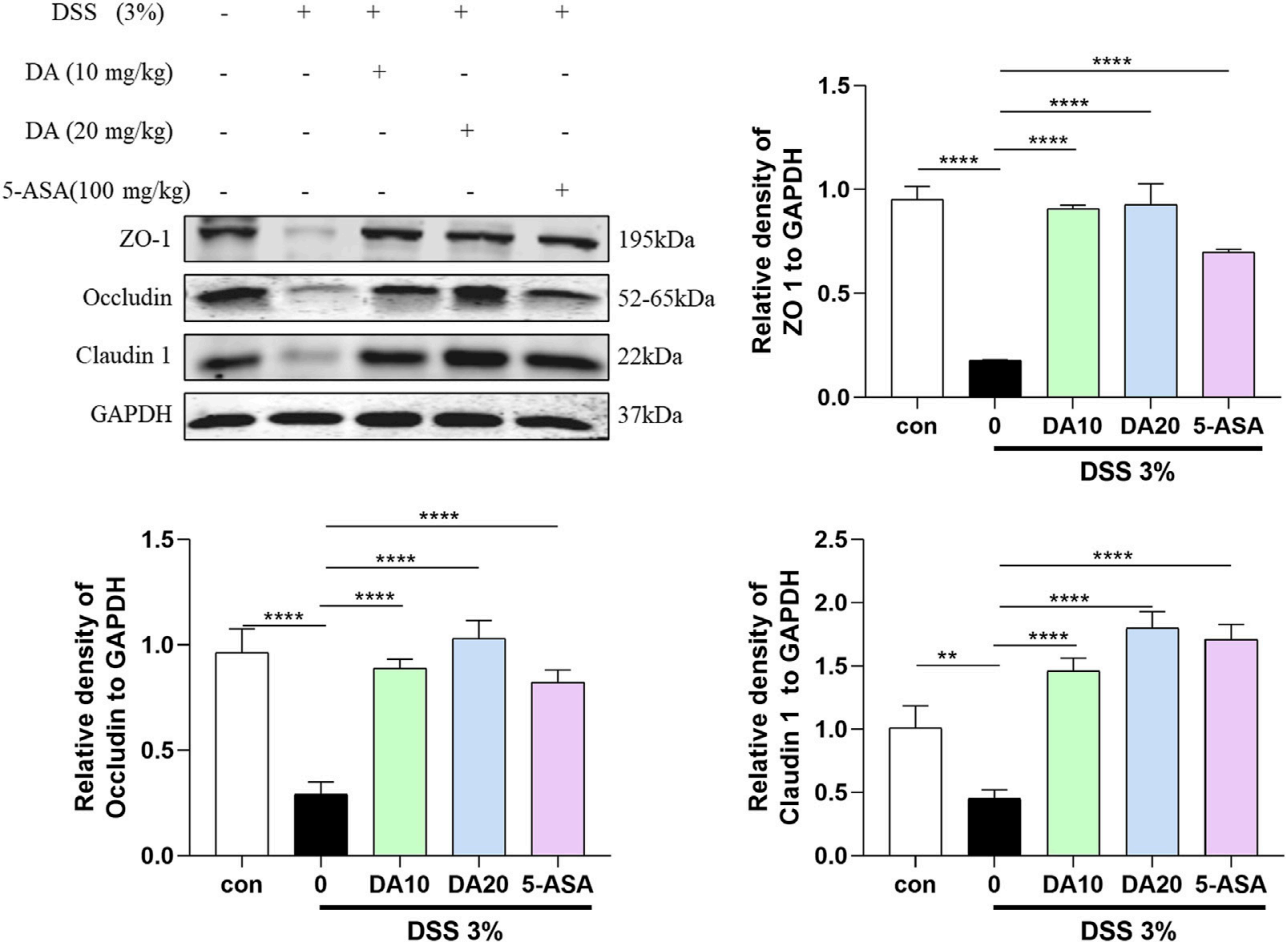
Western blotting and quantitative analysis of ZO-1, Occludin, and Claudin 1 expression in colon tissues in the five treatment groups (n = 3). **P < 0.01, ****P < 0.0001.

Western blotting was also used to analyze the colonic expression levels of the TJ proteins ZO-1, Occludin, and Claudin 1, which are critical for the function of the intestinal barrier. The results showed that the levels of ZO-1, Claudin 1, and occludin were reduced in the colon of mice treated with DSS, and these levels were restored by DA supplementation (Figure 3).

### 3.4 DA inhibits LPS-induced cell death and damage in IEC-6 cells

**FIGURE 4:**
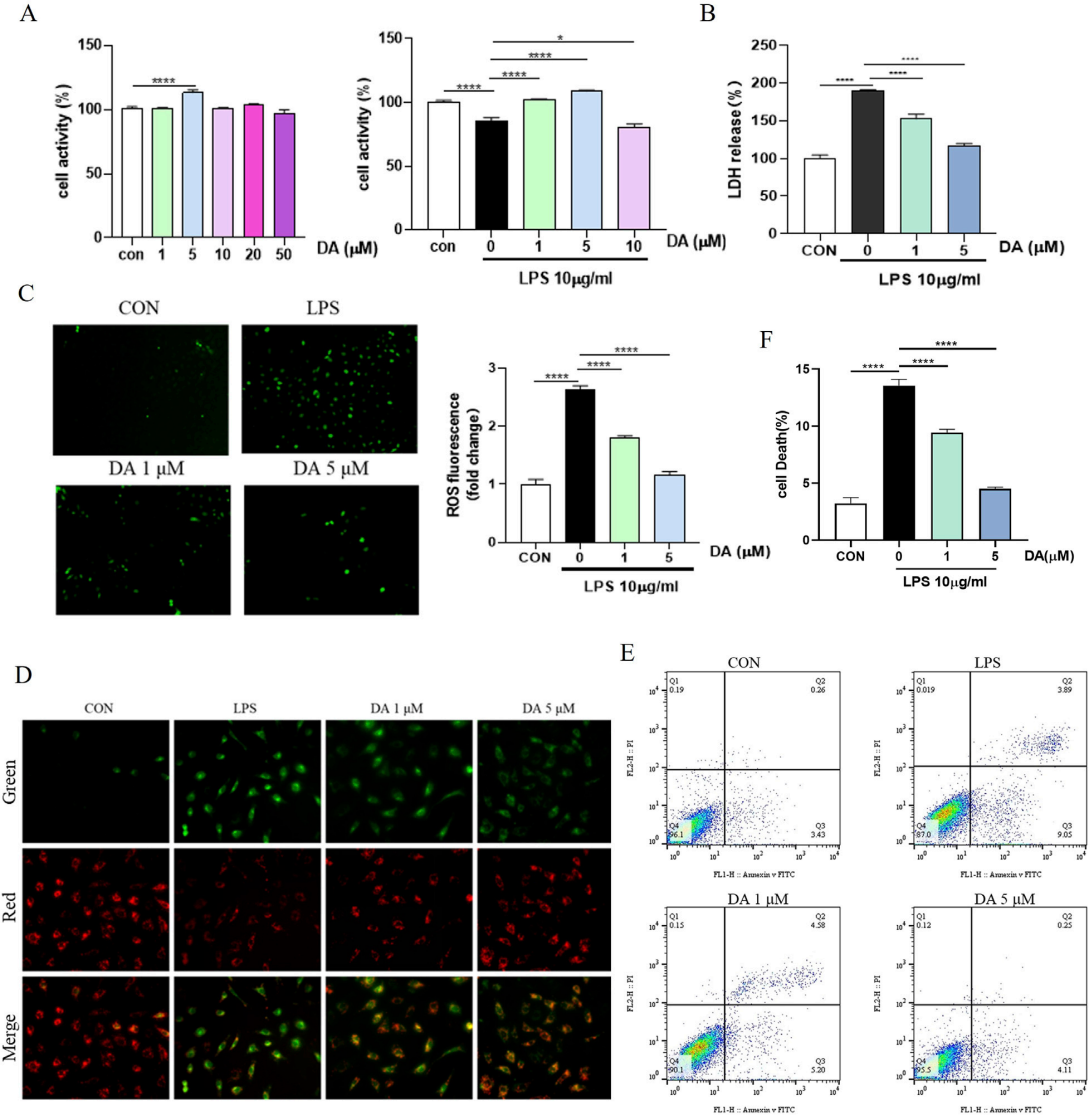
DA inhibits LPS-induced cell death and damage in IEC-6 cells. **(A)** The effective concentration of DA for inhibiting LPS-induced cell damage in vitro by MTT assay. **(B)** Effects of DA on LDH release induced by LPS-induced cell damage. **(C)** Effects of DA on LPS-induced ROS. **(D)** Effect of DA on LPS-induced mitochondrial membrane potential. **(E, F)** Effect of DA on LPS-induced cell death detected by flow cytometry (n = 3). *P < 0.05, ****P < 0.0001.

The results of drug concentration screening by the MTT method are shown in Figure 4A, the maximal noncytotoxic concentration of DA to reach 50 μmol/L in IEC-6 cells, and the effective concentrations for inhibiting LPS-induced cellular damage were 1, 5, and 10 μmol/L, among which 1, 5 μmol/L had the most significant effect. ROS expression levels in IEC-6 cells were measured using the DCFH-DA fluorescent probe assay. As shown in Figure 4C, LPS increased endogenous ROS formation compared to the control group, whereas DA significantly inhibited the expression levels of ROS. The mitochondrial membrane potential was detected by JC-1 as shown in Figure 4D, after LPS induction, the mitochondrial membrane permeability of IEC-6 cells was changed, the green fluorescence was enhanced and the membrane potential was decreased, and after DA administration, the membrane permeability was restored, the green fluorescence was weakened, and the membrane potential was increased. Flow cytometry and LDH assays showed that DA significantly reduced the elevated down-dial mortality produced by LPS (Figures 4B, E, F).

### 3.5 DA inhibits LPS-induced TXNIP/NLRP3 activation and pyroptosis

**FIGURE 5:**
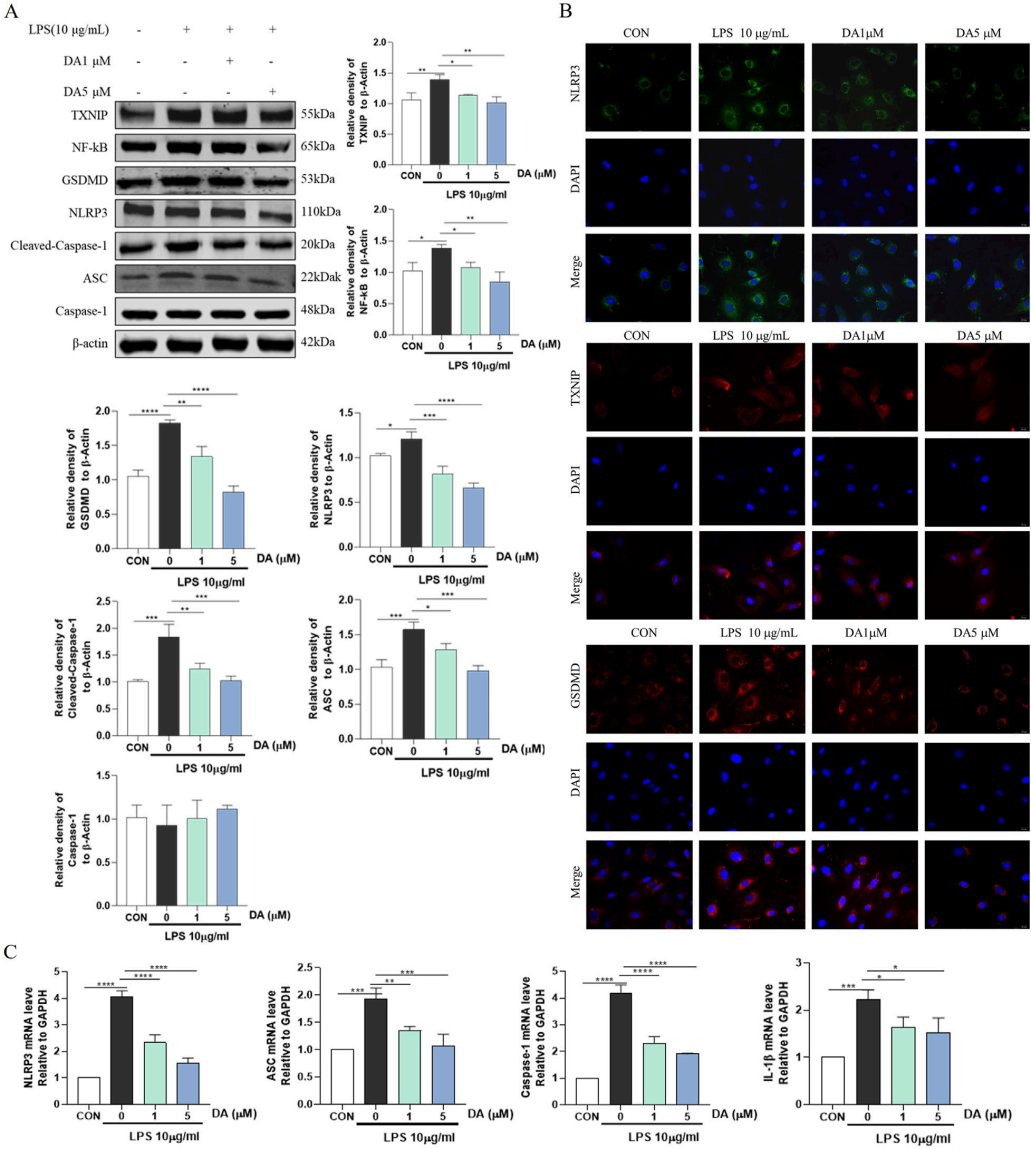
DA inhibits LPS-induced TXNIP/NLRP3 activation and Pyroptosis. **(A)** Western blotting and quantitative analysis of TXNIP/NLRP3 pathway-related protein expression in the cell. **(B)** Immunofluorescence results of the effect of DA on NLPR3, TXNIP, and GSDMD in cells. **(C)** Expression levels of TXNIP/NLRP3 pathway-related mRNA in IEC-6 cells (n = 3). *P < 0.05, **P < 0.01, ***P < 0.001 and ****P < 0.0001.

Western blot results showed that LPS increased the expression of NLRP3, Cleaved-Caspase-1, GSDMD, ASC, NF-κB, and TXNIP proteins in the cells, while DA significantly decreased the expression of these proteins (Figure 5A). Immunofluorescence results showed that the fluorescence intensity of NLRP3, TXNIP and GSDMD was enhanced in LPS-induced cells, which was significantly inhibited by DA (Figure 5B). In addition, LPS upregulated the mRNA levels of NLRP3, ASC, Caspase-1 and IL-1β, which were restored by DA supplementation (Figure 5C). The findings showed that DA reduced the activation of the NLRP3 inflammasome and the pyroptosis brought on by exposure to LPS.

### 3.6 DA upregulates the expression of LPS-inhibited TJ protein

**FIGURE 6:**
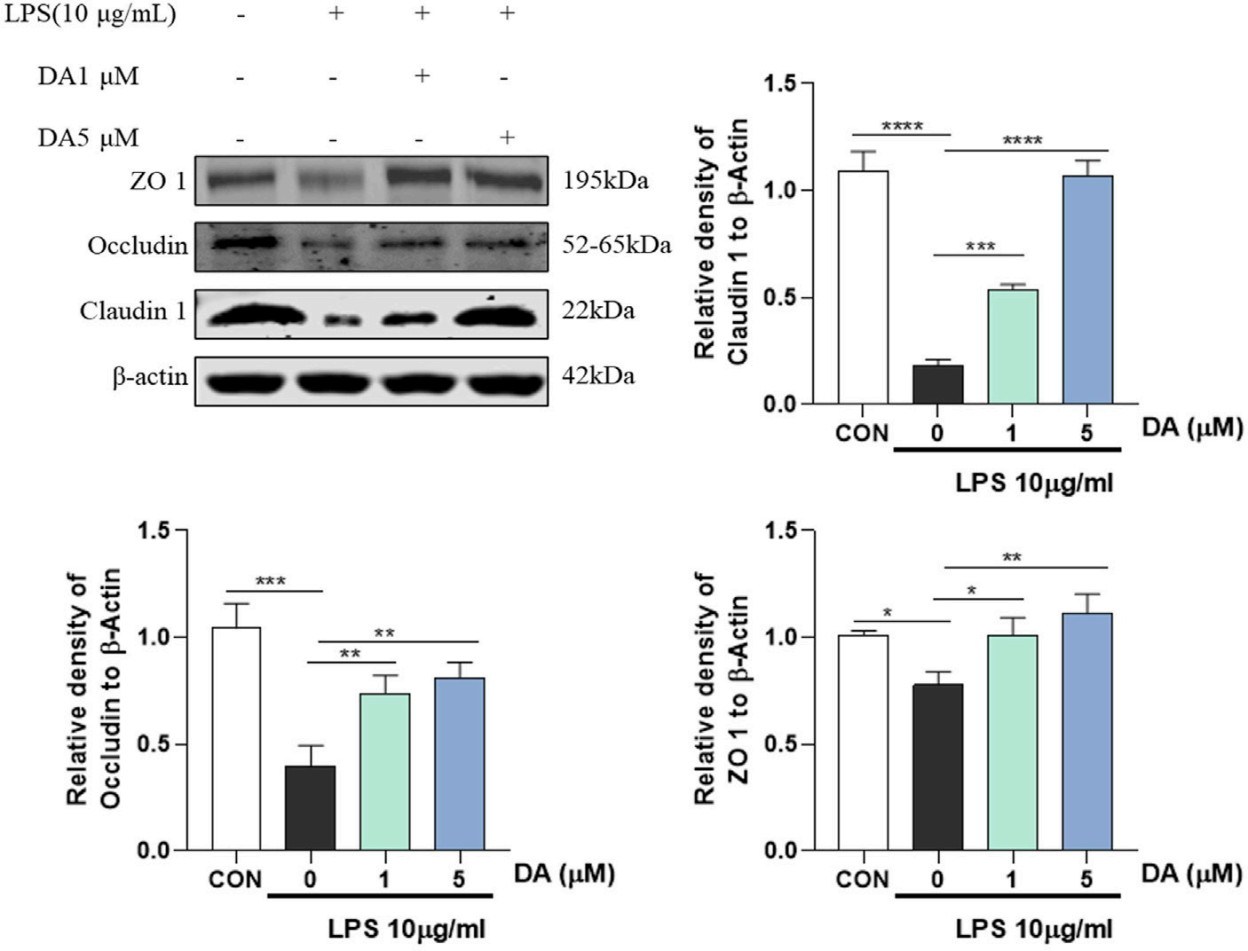
Western blotting and quantitative analysis of ZO-1, Occludin, Claudin 1 expression in cells (n = 3). *P < 0.05, **P < 0.01, ***P < 0.001 and ****P < 0.0001.

It has been reported that NLRP3 inflammasome can promote the expression of IL-1β and IL-18, while LPS and inflammatory factors IL-1β and IL-18 can downregulate the expression or activity of TJ protein (Kaminsky, et al., 2021). As shown in Figure 6, LPS reduced the protein levels of ZO-1, Occludin and Claudin1, and DA treatment significantly increased the protein expression of ZO-1, Occludin and Claudin1 in LPS-stimulated IEC-6 cells.

### 3.7 DA inhibits the NLRP3 inflammasome assembly’s activation

**FIGURE 7:**
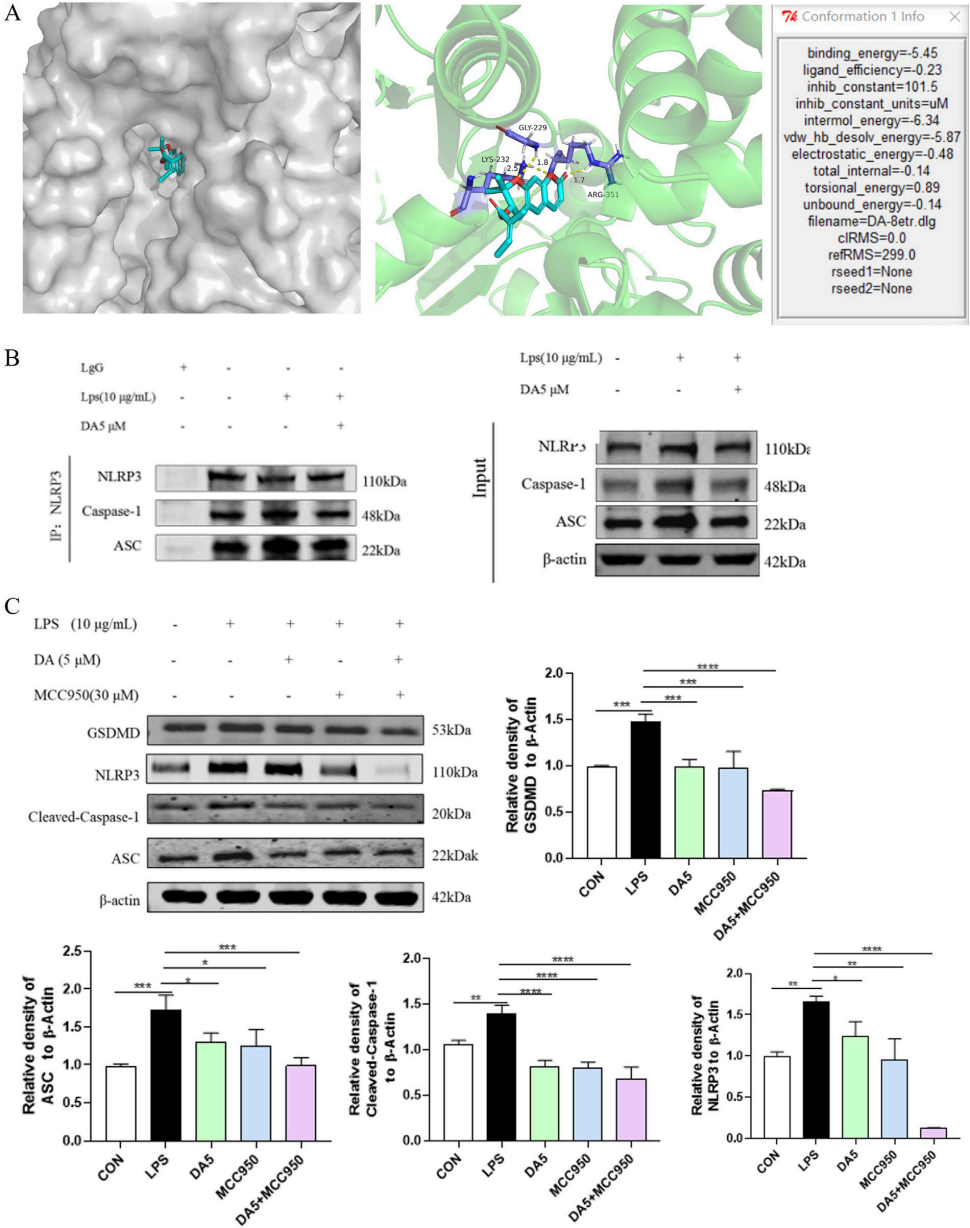
DA inhibits the NLRP3 inflammasome assembly’s activation. **(A)** Mock-up of molecular docking between DA and NLPR3 protein. **(B)** Effect of DA on NLRP3 binding to ASC and Cleaved-Caspase-1. **(C)** Effects of DA and MCC950 on the expression of NLRP3 inflammasome-related proteins (n = 3). *P < 0.05, **P < 0.01, ***P < 0.001 and ****P < 0.0001.

In our investigation into the interactions between DA and the NLRP3 inflammasome, we employed molecular docking simulation techniques to gain a deeper insight into the mechanism by which DA inhibits NLRP3. As illustrated in Figure 7A, the formation of multiple hydrogen bonds between DA and NLRP3 is evident, with a bonding energy of −5.45 kcal/mol, suggesting a strong affinity between the two. In this study, co-immunoprecipitation (Co-IP) was used for validation, and as shown in Figure 7B, LPS significantly enhanced the binding of NLRP3 inflammatory vesicles, whereas DA markedly decreased the interaction between NLRP3 and cleaved-Caspase-1 and ASC, suggesting that DA may influence the assembly of NLRP3 inflammatory vesicles.

We used the NLRP3-specific inhibitor MCC950 as a positive control to verify the molecular docking outcomes (Li et al., 2022). As the results are shown in Figure 7C, LPS increased the protein levels of ASC, GSDMD, NLRP3, and Cleaved-Caspase-1. In LPS-stimulated IEC-6 cells, the augmented protein expression of NLRP3, ASC, Cleaved-Caspase-1, and GSDMD was notably reduced by treatment with DA and MCC950. The expression of these proteins was further suppressed by the combined treatment of DA/MCC950, and the inhibitory effect on the expression of NLRP3 proteins was particularly pronounced.

**FIGURE 8:**
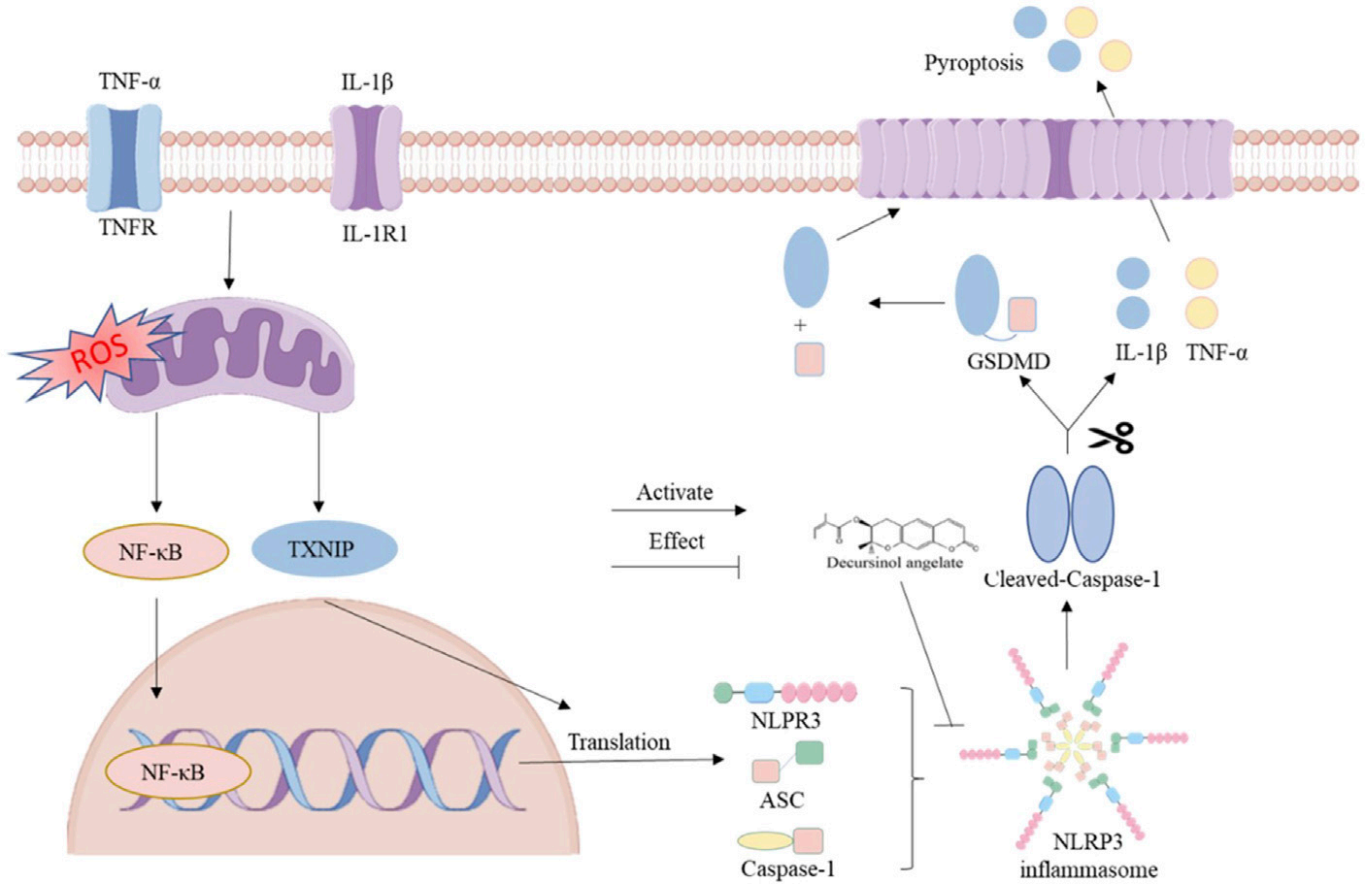
Diagram representing the DA action mechanism for the NLRP3 channel.

The findings suggest that DA has the potential to reduce the inflammatory response by inhibiting the activation of the NLRP3 pathway and the assembly of inflammatory vesicles (Figure 8).

## 4 Discussion

People now understand more about how IBD occurs and progresses because to increased research, and the NLRP3 inflammasome is crucial to this process. NLRP3 inflammasome is one of the potential targets of IBD therapy, especially in the antioxidant process of cells, many processes of inflammatory factor secretion and cell pro-death are associated with NLRP3 inflammasome.

DSS is commonly employed in experiments to induce colitis in mice, and it is widely used in the study of IBD because it induces pathological symptoms that are similar to clinical symptoms (Chassaing et al., 2014). In addition, weight loss and shortened colon reflect IBD severity and shortened colon length may indicate colon dysfunction (Poaty Ditengou et al., 2023). In this study, it was found that mice in the DSS group showed significant weight loss, significantly higher DAI index, and shortened colon length, and the results of the histopathological analysis showed significant rupture of the colonic histological structure and reduction of the mucus layer in the mice, which suggests that the mice developed the pathological features of IBD. The weight loss, colon shortening and DAI elevation of IBD group mice were alleviated after DA treatment, indicating that DA can improve the intestinal physiological state and clinical symptoms of IBD mice.

IBD is primarily driven by oxidative stress, characterized by an imbalance between pro-oxidants and antioxidants (Moura et al., 2020). The pro-inflammatory cytokines TNF-α, IL-1β, and IL-6 are triggered by ROS, which act as signaling molecules attracting and stimulating effector T lymphocyte differentiation. These cytokines are important for controlling intestinal inflammation, immune response, inflammatory cell recruitment, and maintaining chronic inflammation seen in IBD (Abraham et al., 2017). The findings demonstrated that DA considerably reduced the amount of MDA, TNF-α, IL-1β, LPS and IL-6, and increased the content of SOD. This suggests that DA can play a role in alleviating IBD by suppressing the inflammatory response.

Numerous investigations have unveiled the correlation between the NLRP3 pathway and the pathophysiology of IBD. TXNIP expression can rise in response to elevated ROS expression, and both ROS and TXNIP help to activate the NLRP3 inflammasome (Yin et al., 2017). DSS stimulation activates the NLRP3 inflammasome abnormally, which may cause damage to the colon’s mucosa (Bauer et al., 2010). The ASC, caspase-1, and NLRP3 make up the multiprotein complex known as the NLRP3 inflammasome (Ding et al., 2020). Cleaved Caspase-1 is the Caspase-1 subunit that is cut after NLRP3 activation. One of the key regulators of the inflammatory response linked to the etiology of IBD is NF-κB. GSDMD is a crucial component of the two pro-death pathways and a prominent member of the GSDM family (Zuo et al., 2021). The lytic form of cell death known as pyroptosis is brought on by classical or non-classical inflammatory caspase activation. These caspase enzymes lyse GSDMD to produce N-terminal GSDMD fragments, which then undergo pyrosis by creating membrane pores that allow LDH to escape (Rühl et al., 2018). The levels of protein and mRNA transcripts associated with the TXNIP/NLRP3 signaling pathway were significantly elevated in the DSS and LPS group but decreased significantly after DA administration. This suggests that DA can inhibit the NLRP3 pathway and thus play an anti-inflammatory role. The results of this part of the experiment confirmed that DA inhibited the NLRP3 pathway and thus played an anti-inflammatory role.

The intestinal barrier is a physical barrier against foreign substances in the intestinal cavity, and its main component is the mucus layer, which consists mainly of mucins and TJ proteins, transmembrane proteins (claudins, Occludin), and cytoplasmic proteins (Zonula Occludens, ZO) families. An intact mucus layer is an important factor in preventing bacteria from activating the inflammatory response. Beneath the layer of mucus lies the barrier of intestinal epithelial cells, predominantly made up of goblet cells and Pan’s cells. Therefore, repairing mucin and intestinal epithelial compact junction protein and improving intestinal barrier function are potential research strategies for the treatment of IBD. In this study, AB staining was employed to assess the integrity and mucin content of the mucus layer and the results showed that in the DSS group, the intestinal mucosal barrier was disrupted, resulting in a reduction of mucin and a decrease in the mucus layer. In addition, Western blot detection of related proteins showed that the levels of ZO-1, Occludin and Claudin 1 proteins were significantly reduced in both *in vivo* and *in vitro* models, whereas DA could upregulate the expression of the above proteins. Therefore, the study revealed that DA was capable of restoring the integrity of the intestinal mucosal barrier in mice with IBD, thereby providing a protective role in the gastrointestinal tract.

In this study, LPS-induced IEC-6 cells to establish an *in vitro* injury model. IEC-6 cells are indispensable for acquired immunity and mucosal innate defense mechanisms and are often used as models of intestinal diseases *in vivo* and *in vitro*. The primary constituent of the outer membrane of gram-negative bacteria, known as LPS or endotoxin, is what causes the inflammatory response associated with IBD (Li et al., 2018), causes inflammatory damage to IEC-6, and causes pro-death of cells, which is used to explore the pathological mechanism of various diseases. Initially, MTT was employed to assess cell viability expression when exposed to varying concentrations of DA, and the optimal concentrations of DA (1 μM and 5 μM) were determined, and subsequent cell experiments were conducted with these concentrations. LPS-induced IEC-6 cells to establish an inflammatory injury model *in vitro*. Inflammation is often accompanied by mitochondrial damage and overproduction of ROS. Tissue damage during inflammation produces free radical ROS, which has harmful effects on cell function (Luo et al., 2024). The cell damage also leads to a large amount of LDH release. In this study, after treatment with LPS, cells showed elevated levels of ROS, decreased mitochondrial membrane potential, increased mortality, and increased levels of LDH release, which were dose-dependently inhibited by DA. These results suggest that DA can repair LPS-induced cellular damage.

The precise chemical mechanism between DA and NLRP3 was confirmed to delve deeper into the regulation mechanism of DA on the NLRP3 inflammasome. One popular virtual screening technique for predicting the binding conformation of tiny molecular ligands with appropriate target binding sites is molecular docking (Dong et al., 2018). An example of a distinct small molecule inhibitor capable of selectively blocking NLRP3 inflammasome activation is MCC950 (Wu et al., 2020). One of the common techniques for determining or validating protein interactions within the body is Co-IP. Binding of target protein-specific antibodies to affinity beads to identify drug interactions with specific proteins (Lin and Lai, 2017). The results showed that DA had a good affinity with NLRP3. Western blot results showed that MCC950 and DA expressed the same inhibitory effect, and there were significantly more ASC and Cleaved-Caspase-1 bound to NLRP3 when induced by LPS. After immunoprecipitation of NLRP3 protein, DA significantly inhibited the expression of ASC and Cleaved-Caspase-1, and the binding of NLRP3 to ASC and Cleaved-Caspase-1 was reduced. These results suggest that DA may reduce the activation of NLRP3 inflammasome assembly and inhibit the activation of the NLRP3 pathway through binding to NLRP3 protein, thus exerting its anti-inflammatory effect.

We can suggest potential DA therapy strategies for IBD based on the aforementioned findings. First, DA inhibits oxidative stress, inhibits the secretion of pro-inflammatory factors, regulates the ROS/TXNIP/NLRP3 pathway, maintains intestinal barrier homeostasis, and improves intestinal dysfunction (Figure 8). However, the experimental validation of the mechanism in this study is relatively single, and other methods are needed to further validate and explore the mechanism and to fully elucidate its pharmacodynamics. Therefore, in future studies, we will use DA as the basis to further explore its mechanism of action in alleviating IBD.

## 5 Conclusion

Our findings demonstrate that DA has the potential to restore the integrity of the injured intestinal mucosa, alleviate intestinal inflammation, and mitigate the physiological symptoms associated with IBD. Additionally, DA appears to counteract oxidative damage.

The mechanism by which DA exerts its effects on IBD involves the repair of the intestinal barrier function. This is achieved through the inhibition of inflammatory factor secretion, activation of the NLRP3 inflammasome, and suppression of the ROS/TXNIP/NLRP3 signaling pathway. In summary, our research provides novel insights into the therapeutic actions of DA in treating IBD and advocates for its broader application in pharmacological treatments.

## Data Availability

The original contributions presented in the study are included in the article/supplementary material, further inquiries can be directed to the corresponding author.
